# Utilization of Immersive Virtual Reality in Cognitive Stimulation Therapy (IVR-CST) for elderly with mild cognitive impairment: A randomized controlled pilot study protocol

**DOI:** 10.1371/journal.pone.0330686

**Published:** 2025-08-26

**Authors:** Winsy Wing Sze Wong, Gloria Hoi Yan Wong, Jacky Chak Pui Choy, Donald Shi Pui Li, Yee Lam Lo

**Affiliations:** 1 Department of Chinese and Bilingual Studies, Faculty of Humanities, Hong Kong Polytechnic University, Hunghom, Kowloon, Hong Kong SAR, China; 2 School of Psychology and Clinical Language Sciences, University of Reading, Reading, United Kingdom; 3 Department of Social Work and Social Administration, Faculty of Social Sciences, The University of Hong Kong, Pokfulam, Hong Kong SAR, China; 4 Department of Cognitive Science, Krieger School of Arts and Sciences, Johns Hopkins University, Baltimore, Maryland, United States of America; PLOS: Public Library of Science, UNITED KINGDOM OF GREAT BRITAIN AND NORTHERN IRELAND

## Abstract

**Objectives:**

Mild cognitive impairment (MCI) affects about 11.4% of the elderly population in Hong Kong. This study mainly investigates the feasibility and efficacy of immersive virtual reality-based cognitive stimulation therapy (IVR-CST) on MCI, and the use of eye-tracking technology in studying treatment outcome.

**Hypothesis to be tested:**

1) Whether IVR-CST is a feasible intervention for the elderly with MCI. 2) Whether IVR-CST is efficacious (and more efficacious than conventional CST) in improving cognition. 3) Whether changes in eye movements across therapy and treatment outcome are associated.

**Design and subjects:**

An open-label, two-armed, assessor-blinded, randomized controlled trial will be conducted. Sixty-six elderly individuals with MCI will be recruited and randomly allocated to either the IVR-CST or the conventional CST group. Their cognition will be measured before and immediately after therapy and 4 weeks post-therapy.

**Interventions:**

A 14-session IVR-CST or conventional CST, with content adapted from the Chinese-translated manual of CST, will be carried out twice per week in groups of three to four individuals.

**Outcome measures and data analysis:**

The Hong Kong Montreal Cognitive Assessment and measures on executive functions/working memory will serve as primary outcomes. The within-subject (before and after therapy) and between-subject (IVR-CST vs. conventional CST) differences will be examined. Besides, eye movements during therapy in the IVR-CST group will be collected and its correlation with primary outcomes will be studied.

**Expected results:**

Positive changes in cognition are expected after therapy in both treatment groups, which may be maintained four weeks post-therapy.

**Trial registration:**

ClinicalTrials.gov ID: NCT06838494

## Introduction

MCI, a mild neurocognitive disorder as defined by the Diagnostic and Statistical Manual Fifth Edition (DSM-5) and Word Health Organization – International Classification of Diseases (WHO-ICD), affects various cognitive domains such as memory, attention, language, executive functions, and visual-spatial processing but yet the individual’s functions in daily activities are relatively normal. MCI is regarded as a heterogeneous condition between normal aging and dementia [1]. The prevalence of MCI, which may vary across countries, cultures, and age, is reported to range from 3% to 42% [2]. Whereas in Hong Kong, the incidence rate is about 114.4 cases per 1000 person-years [3]. Individuals with MCI have a much higher chance of developing dementia when compared to those without MCI. It is estimated that 46% of the MCI population will convert to dementia when compared to a 3% chance of those without MCI [4]. Much effort has been devoted to improving the early identification and management of MCI. Most of the intervention studies investigated remedies that have been tested in the dementia population. Pharmacological interventions studied the use of cholinesterase inhibitors, galantamine, and donepezil, while nonpharmacological interventions investigated the effects of behavioral programs (e.g., physical or cognitive exercises) and non-invasive brain stimulation (e.g., repetitive transcranial magnetic stimulation) on cognitive functions of people with MCI. Findings from systematic reviews revealed that pharmacological interventions are not beneficial to cognition [1,5], and a small but uncertain cognitive benefit has been found in behavioral treatment [5,6]. The conclusion on the effects of non-invasive brain stimulation has remained inconclusive [7]. Further research on non-pharmacological interventions with high-quality randomized controlled trials (RCTs) is encouraged.

### Cognitive stimulation therapy: A potentially beneficial intervention for MCI?

Despite the lack of official guidelines for treatment on MCI, recommendations from institutes/organizations on the management of dementia may shed light on directions for future development. Non-pharmacological interventions are considered the key component to managing the disease. Among the three approaches to cognitive intervention on dementia defined by Clare and Woods, cognitive stimulation, as defined as “engagement in a range of activities and discussions (usually in a group) aimed at general enhancement of cognitive and social functioning”, has been regarded as beneficial to people with dementia (PwD) [8]. Three key aspects of cognitive stimulation, namely ‘generalized cognitive exercise’, ‘social interaction’, and ‘social support’, are suggested to constitute the underlying mechanisms leading to treatment success [9]. A recent Cochrane Review concluded that cognitive stimulation has a small and short-term benefit (a standard mean difference of 0.43 s.d.) on the general cognitive functioning of people with mild-to-moderate dementia [9]. Contrastively, the other two approaches, namely cognitive training, which involves drilling of decontextualized and standardized tasks tapping certain cognitive processes, and cognitive rehabilitation, which refers to use of strategies to accomplish daily functions, are much less effective (or even not effective when cognitive training is considered) in promoting cognition of PwD [10]. Among different cognitive stimulation programs, CST, an intervention with high-quality evidence based on RCTs, is considered a more popular and influential group intervention protocol with much research support from a worldwide perspective [11]. CST, a 14-session group-based activity program for mild-to-moderate PwD, is conducted by one or two trained facilitators (who can be a health care professional or a volunteer) and a group size of five to six PwD at most. A total of 18 key principles in implementing CST, e.g., mental stimulation, stimulating language and executive functioning, and building/strengthening relationships, are emphasized in facilitator training and CST manuals. In each session, different themed activities such as childhood, current affairs, and physical/word/number games are carried out to provide generalized cognitive exercise to the participants. Furthermore, aspects of social interaction and support are achieved via frequent and continual interpersonal communication in a group context throughout therapy. CST has been recommended by the National Institute for Health and Care Excellence (NICE) of the United Kingdom government [10] and the World Alzheimer Report of ADI (2011) for PwD. CST has been available in at least 34 countries, while in a local context, a translated manual in Chinese [12], a pilot study on conventional CST [13], and a preliminary virtual individual CST (vCST) study delivered via Zoom have been conducted [14] with positive changes in cognition demonstrated in both studies. In view of the success CST has brought to PwD, it is reasonable to hypothesize that individuals with MCI may potentially benefit from it. Since the presenting symptoms and neuropathology of MCI and dementia are highly similar [15], the mechanisms accounting for the cognitive improvements induced by CST would likely take place in the MCI population. In fact, an RCT, based on adapted CST activities, has demonstrated positive effects on global cognition (with a medium effect size of d=0.564) in terms of the Spanish version of MMSE [16]. Positive as it may seem, one may question if the level of difficulty of the themed activities offered in CST may be too easy or not mentally stimulating to the MCI population. The present study intends to provide a solution via the application of immersive virtual reality (IVR) – a novel and safe means to create a stimulating and novel therapy context, and to take advantage of the technological advancement IVR offers in monitoring of users’ experience to facilitate treatment delivery and enhance treatment outcome.

### IVR-CST: Justifications and potential benefits for MCI

VR has been applied in health care and therapy delivery for more than 20 years. It aims to create an artificial environment to replace real-world surroundings via equipment such as a head-mounted display (HMD), headphones with music/sound, and devices for manipulation/navigation in the virtual environment. The application of VR/IVR in MCI/dementia therapy has been emerging. A recent systematic review studying VR-based neuropsychological interventions reported a moderate effect and a large effect on global cognition (g = 0.30) and executive functions (g = 0.60) of older adults with MCI (mean age about 70 years), respectively [17]. More specifically, another review [18] compared the efficacy of VR-based cognitive rehabilitation with those without VR, in which IVR was used in one of the studies [19]. VR-based intervention was at least as effective (or more effective than conventional treatment without VR in some of the outcomes) as conventional cognitive rehabilitation on the MCI population [18]. In addition, good user acceptability, minimal cybersickness/adverse events, and preference for IVR have provided further incentives and support to future research with well-designed RCTs [20]. As a matter of fact, a closer look at the studies included in the review on VR may shed light on issues related to study design and theoretical considerations. Firstly, regarding the nature of intervention, most of the studies belonged to cognitive rehabilitation, an intervention approach less recommended by NICE for PwD. In contrast, the efficacy of VR-based cognitive stimulation has rarely been studied, except for pilot studies [21]. Secondly, little was known about the theoretical basis for the selection of treatment content/protocol. Most of the studies argued that multisensory experience and a more focused environment rendered by IVR might promote treatment success. Little consideration/attention was made regarding how the treatment per se, and its underlying mechanism, may improve the cognitive functions of the participants. If IVR is applied to treatment protocols with uncertain treatment efficacy/mechanism accounting for treatment success, its power in promoting treatment outcomes will be undermined. To address the above issues, our proposed study aims to incorporate IVR in CST delivery and investigate its efficacy in improving the cognition of people with MCI with the following justifications. Firstly, CST is a well-developed and evidence-based program; its implementation in the local MCI population is culturally appropriate and ecologically valid. Based on a case series conducted by the first author, the use of IVR-CST yielded positive results in terms of users’ acceptability, compliance, and some improvements in communication among PwD with visual impairment [22]. Secondly, during IVR-CST, users will immerse themselves in a novel virtual environment and engage in a series of cognitively stimulating activities, which are concurrent with the principles of CST, such as mental stimulation, being fun, and choice. Thirdly, the immersive environment may enable the participants to focus and pay better attention to the treatment stimuli. Last but not least, social participation and connectedness, which are important principles of CST, are readily provided during group interactions.

### Eye-tracking in IVR: A biomarker for treatment response?

The application of IVR may go beyond from treatment delivery to investigation of treatment response. Recent technological advancement has enabled reliable and accurate eye-tracking in commercially available HMDs, which allows researchers to explore such applications in theoretical and clinical perspectives. Eye-tracking is a technology which captures distinct eye movement types. For example, in saccadic eye movements in which the eyes scan the visual field via successive eye movements, the eyes will fixate on a certain area of interest for 200–300 ms before moving again. Hence, eye movements such as the position, number, and duration of fixations can be traced via an eye-tracker. According to a recent review on applications of eye-tracking in VR, some of the clinical applications included diagnosis and treatment of neurodegenerative disorders, measurement on visual attention, evaluation of cognitive effort, and as well as evaluation of learning outcomes [23]. Eye-tracking can be regarded as an effective way to detect cognitive decline in people with dementia/MCI [24] As such, eye-tracking seems to be a viable means to investigate the eye movements of PwD/ people with MCI during VR-based therapy, e.g., to examine whether the participant is paying enough attention to the details of the stimuli presented. Furthermore, based on previous studies on healthy adults that the average duration of fixation and the number of revisits successfully predicted learning outcomes [23], one may question if there is a causal relationship between changes in eye movements and treatment gain in MCI population. Nevertheless, this hypothesis has rarely been investigated.

### Aims and hypotheses to be tested

The proposed study aims to investigate the potential of IVR-CST in improving the cognition of people with MCI. Three research questions have been proposed and listed as follows:

1. Is IVR-CST a feasible treatment for the elderly with MCI?
2. Is IVR-CST an efficacious treatment for improving cognition in the elderly with MCI?
3. Is IVR-CST more efficacious than conventional CST (i.e., without IVR) in improving the cognition of the elderly with MCI?
4. Is eye-tracking data collected during therapy associated with treatment outcomes of IVR-CST?

## Materials and methods

### Study design

**Fig 1:**
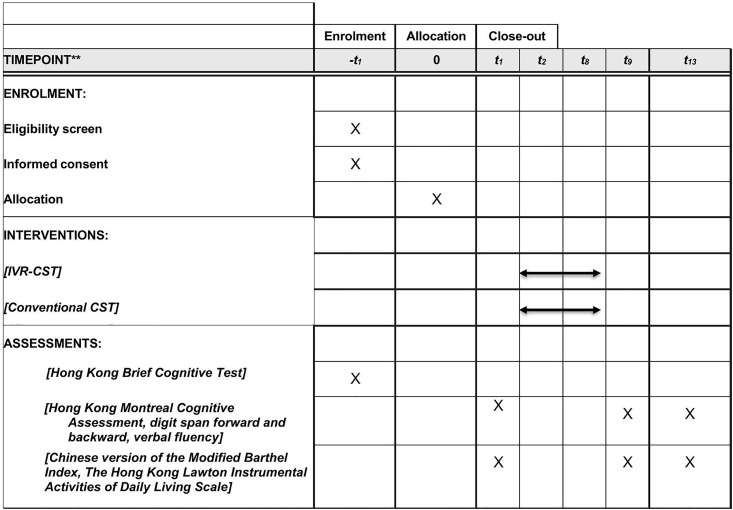
SPIRIT schedule. SPIRIT schedule illustrating the study design.

An open-label, two-armed, assessor-blind, randomized controlled trial will be conducted. Randomization will be done by the principal applicant using a computer-generated random sequence. Participants will be randomly allocated to the IVR-CST or conventional CST group. The assessor will be blinded to condition allocation. Both the assessor and the participants will be reminded not to talk about issues related to treatment when they meet. The treatment protocol (version 1.0, dated March 3, 2025), which contains items from the World Health Organization Trial Registration Data Set, has been registered in ClinicalTrials.gov (NCT06838494). We will inform the funding agency, the Institutional Review Board of the Hong Kong Polytechnic University, ClinicalTrials.gov, and the publisher of the protocol of any amendments to the protocol. The experimental design is summarized in Fig 1.

### Participants

A total of 66 community-dwelling participants with MCI will be recruited from community centres for the elderly and Non-Governmental Organizations (NGOs) providing care services to elderly people with cognitive impairment in different districts in Hong Kong. The proposed sample size is calculated using G*power 3.1.9.4, based on a power estimate of 80% with α = .05, an effect size of d = 0.40 (with reference to the averaged effect size of 0.43 on global cognition, as reported in the latest Cochrane Review [9] on cognitive stimulation on PwD), and an attrition rate of 25%. The effect size of cognitive stimulation on PwD ranged from 0.3 to 1.32 [9], while an effect size of 0.564 was reported in adapted CST on MCI [16]. Also, the effect size of previous RCTs using VR on cognitive intervention of MCI ranged from 0.6 to 0.9 on measures of general cognition. Thus, an effect size estimate of 0.4 for the current proposal is considered relatively conservative. The attrition rates of previous VR studies on MCI are considered low (6–14%); thus, the estimated attrition rate of the current proposal is considered modest.

Inclusion criteria include: 1) elderly aged 60 years or above. 2) A score of 16–21 out of 30 in the Hong Kong Brief Cognitive Test [25], with upper and lower cutoff scores for MCI as suggested by the test. 3) ability to speak and comprehend Cantonese; 4) normal or corrected-to-normal vision and hearing; 5) absence of physical illness/disability to prevent them from IVR-CST participation; and 6) compatible with IVR exposure in the 10-minute IVR trial without major signs of cybersickness, based on the symptoms given in the Simulator Sickness Questionnaire [26]. Exclusion criteria include 1) concurrent participation in other clinical therapy trials; 2) incompatibility with IVR exposure such as complaints of nausea, headache, or other severe discomforts during trial use; 3) a diagnosis of dementia or other psychiatric/neurological diseases such as depression, stroke, brain trauma, Parkinson’s disease; 4) hearing/visual/upper limb impairments that hinder CST/IVR-CST participation; 5) prior CST treatment; 6) Use of medication for MCI/dementia, e.g., aducanumab. Screening will be done by a trained research assistant in a quiet room. Participants’ demographic information, medical and family history will be obtained.

### Randomization

Subjects who have given informed consent will be randomly allocated to either IVR-CST or conventional CST condition, based on a computer-generated randomization sequence created by one of the co-authors (Li) with an allocation ratio of 1:1.

### Assessment and treatment schedule

All participants will be assessed at three time points throughout the study: week 1, week 9, and week 13 to evaluate their performance before, immediately after, and in the maintenance phase, respectively. The treatment schedule will be the same for both treatment conditions, in which 14 therapy sessions will occur twice per week from week 2 to week 8.

### Outcome measures and procedures

Primary outcomes: 1) The Hong Kong Montreal Cognitive Assessment (HK-MoCA; 27). It is a sensitive cognitive measure for MCI [27] and covers different cognitive domains, including visuo-spatial skills, executive functions, attention, memory, language, and orientation. The total score (out of 30) will be used to reflect global cognition; 2) Digit span forward and backward tasks, measures of working memory that predict progression from MCI to dementia [28]; 3) Verbal fluency of semantic category. Verbal fluency, which reflects semantic integrity, cognitive flexibility, and executive function, serves as a sensitive task in detecting MCI [29] and predicting conversion to dementia [30]. The number of items correctly produced in one minute in different semantic categories (animals, fruits, and transportation) will be tested.

Secondary outcomes: 1) Chinese version of the Modified Barthel Index [31], which assesses one’s level of independence in activities of daily living such as grooming, feeding, mobility, toileting, etc.; 2) The Hong Kong Lawton Instrumental Activities of Daily Living Scale [32], which evaluates the level of independency in performing community-living skills including cooking, household management, shopping, etc.

Feasibility outcome and users’ experience: Objective and quantifiable feasibility outcomes, including the number of adverse events and technical issues that occurred during intervention, and the level of adherence (actual duration and number of sessions conducted vs. scheduled) to the intervention protocol of both treatment groups, will be measured. Participants of the IVR-CST group will also attend a 20-minute semi-structured interview conducted post-therapy on topics including levels of comfort, presence of adverse effects such as cybersickness, and impact of therapy on daily functions.

Eye-tracking data obtained from the IVR-CST group will serve as an exploratory outcome. In each of the sessions, areas of interest (AOI) of the stimuli presented will be identified. For example, the childhood snacks or toys presented in the themed session ‘Childhood’ will be regarded as AOI. Parameters, including the number of fixations, duration of fixation, and number of revisits on the AOI generated by each participant, will be recorded in the HMD.

The above-mentioned assessment will be done by a trained research assistant with a healthcare background (e.g., rehabilitation science) who has no knowledge of the therapy condition of the subjects, and the whole process will be audio-taped.

### Treatment materials and procedures

In the IVR-CST group, a total of 14 IVR-CST sessions will be carried out twice per week, with each session lasting about 45 minutes. Therapy will take place in a quiet room in the community elderly centres. A research personnel who is a healthcare professional (e.g., occupational or speech therapist) with a certified CST facilitator qualification will be the group facilitator, and three to four participants with MCI will receive IVR-CST as a group throughout the sessions. An assistant will also be present to provide help if necessary. Treatment instruments will include commercially available HMDs with eye- and hand-tracking functions available (e.g., Meta Quest Pro) for each participant. Treatment materials (i.e., content to be delivered in each themed session) will be adapted from the Chinese-translated manual of CST [11]. Materials presented via HMD will include commercially available VR games suitable for the elderly, photo-real panoramic (i.e., 360-degree) images or videos, and two- or three-dimensional animated images and photos. Use of hand controllers or hand movement will be allowed in some activities, such as physical games or manipulation of 3D objects. Similar to the original CST manual, two activities with varying levels of difficulty will be available to allow some flexibility. The major difference between the level of difficulty of the currently proposed vs. the existing manual lies in the complexity of the tasks or the use of less familiar stimuli. For example, in session 10 ‘Orientation’. The original manual suggests the use of familiar local places as stimuli for discussion; in IVR-CST, less familiar places like overseas tourist spots will be introduced. Sample themed activities are given in Table 1. Treatment procedures will follow the Chinese-translated manual of CST [12]. In the first 10 minutes, participants and the facilitator will greet and meet, and sing-along a group song or share some snacks/drinks. Reality orientation will be followed by telling the participants the time, weather, and recent news/affairs. Then the facilitator will assist the participants to wear the HMD for about 30 minutes, during which the themed activity will be conducted. All participants will be seated comfortably and at a distance far enough from one another to ensure safety during hand movement during IVR exposure. According to the IVR-CST manual, the facilitator will guide the participants to conduct a range of activities, discussions, or sharing. Material presentation will be controlled by the facilitator via a laptop. S/he will not wear an HMD so that immediate assistance to the participants can be provided. In the last five minutes, the HMD will be taken off while the facilitator will round up the session. In the conventional CST group, the same treatment activities, materials, and procedures will be used, while photos, pictures, or real objects will be presented.

**Table 1:** Examples of themed activities of IVR-CST and conventional CST.

Session	Theme	IVR-CST	Conventional CST
1	Physical games	Level 1: IVR games requiring simple hand movement (e.g., fishing, ball throwing) Level 2: IVR games requiring more complex hand movement (e.g., coordination of both hands, playing darts)	Level 1: Tabletop games requiring simple hand movement (e.g., ball throwing to play tic-tac-toe) Level 2: Tabletop games requiringmore complex/refined upper limb movement (e.g., playing darts)
2	Sound	Level 1: Guessing game in which unfamiliar sounds or songs will be played. Level 2: Music sandbox game to create a song/rhythm via playing different musical instruments via limb detection/hand controller via VR.	Level 1: Guessing game in which unfamiliar sounds or songs will be played. Level 2: Music sandbox game to create a song/rhythm via playing different musical instruments provided onsite.
6	Faces/scenes	Level 1: Showing of photos/videos of old places/ celebrities via IVR for guessing, comparison, and discussion. Level2:Showing childhood/teenage photos of the participants via IVR for guessing, comparison and discussion.	Level 1: Showing of photos/videos of old places/ celebrities for guessing, comparison, and discussion. Level2:Showing childhood/teenage photos of the participants for guessing, comparison and discussion.
9	Categorizing objects	Level 1: Verbal fluency of different concepts/ semantic categories with cues provided via IVR. Level 2: Object categorization using different strategies with cues provided via IVR.	Level 1: Verbal fluency of different concepts/ semantic categories Level 2: Object categorization using different strategies

This table summarizes a few themed activities of the IVR-CST and conventional CST sessions.

### Data analysis

Primary and secondary outcomes will be scored by a trained research assistant who is not involved in the assessment. To further reduce observer bias, a random code will be given to the scoring forms and audio-recording so that the research assistant will be blind to the identity of the participant, the experimental group assigned, and the timepoint of assessment. A linear fixed-effect model will be used to evaluate the participants’ performance in the outcome measures, as this statistical model is better in handling repeated observations, missing data, and individual variability [33]. Both time points (week 1/9/13) and conditions (IVR-CST vs. conventional CST) will serve as fixed factors, and scores of primary/secondary outcomes as dependent variables. Participants’ characteristics, such as age, baseline cognitive performance, and education, will be included in the model as covariates. Any significant main effects or interactions will be investigated via post-hoc analyses. Measurements concerning feasibility and user experience will be evaluated descriptively. In addition, a narrative summary of responses collected during the post-therapy interview will be reported.

For exploratory outcome, a 5-minute sample will be extracted from each participant and session from the IVR-CST group. To study possible changes in eye movement across sessions, the aforementioned eye-tracking parameters, including number of fixations, duration of fixation, and number of revisits on the AOI generated by each participant, will be evaluated by linear fixed-effect models as dependent variables, with session number as a fixed factor. Any significant main effects will be followed by post-hoc analyses. To investigate the possible associations between eye movement and treatment outcome, the correlation between the averaged difference in number and duration of eye fixations of each participant in the first and last three IVR-CST sessions and the difference score of HK-MoCA before and immediately after therapy will be analysed. If the correlation reaches statistical significance, multiple regression will be conducted, with the eye-tracking parameters as predictors, while the difference score of HK-MoCA pre/post therapy will serve as the predicted variable.

### Safety considerations

Exposure to VR is generally considered to be safe. Nonetheless, a few precautions will be taken to ensure the subject’s safety and acceptance. Firstly, in the screening session, each participant will have a 10-minute IVR exposure in which s/he will experience the visual/auditory input and perform gestures/actions similar to those used in the therapy sessions. Those incompatible with the IVR exposure will be excluded from the study. Secondly, participants will be seated in armchairs at a safe distance from one another to ensure safe navigation during IVR-CST.

### Reliability and treatment fidelity

About 10% of the data of primary outcomes will be randomly selected and blindly scored by the research assistant and an independent research assistant to examine intra- and inter-rater reliability, respectively. Point-to-point agreement will be used. Treatment fidelity will be assessed by an independent certified CST trainer. The latter will be invited to observe seven of the treatment sessions selected at random. A checklist compiled in accordance with the recommendations given by the National Institute of Health Behaviour Change Consortium will be used [34].

### Ethics statement

Ethical approval has been obtained from the Institutional Review Board of the Hong Kong Polytechnic University (HSEARS20241010009) on 11 December 2024. A trained research assistant will explain the aims and procedures to the participants and their carers before written consent is sought. Subjects have the right to withdraw from the study without any consequences.

### Timeline of the study

The whole study period will span from July 2025 to October 2026. Participant screening and recruitment will be conducted from July to October 2025, while intervention and outcome measurement will take place from November 2025 to July 2026.

### Data management plan

Data management is handled by the Principal Investigator of the project (the first author) and the co-authors of the protocol, which is compliant with the requirements of the Institutional Review Board of the Hong Kong Polytechnic University and independent from the funder. Paper sources will be stored in locked file cabinets, which are only available to the research team members. Audio recordings will be stored on secure servers provided by the Hong Kong Polytechnic University and are only accessible by the research team members. Data shared on an open-access repository will be de-identified via a unique subject code identifier before distribution. Any incident concerning unauthorized data access will be reported to the Institutional Review Board of the Hong Kong Polytechnic University.

## Discussion

The current study protocol aims to investigate the feasibility and clinical efficacy of IVR-CST on people with MCI. The findings will inform us about the acceptability of VR among people with MCI, the suitability of applying VR in cognitive therapy, and its efficacy when compared to conventional therapy protocols targeting the MCI population. Feedback collected from users with MCI allows clinicians, engineers, and researchers to better understand users’ perception in using VR, which is critical for the better design of hardware/software systems and content delivery for therapeutic contexts.

There are a few limitations of the proposed study. Firstly, the small sample size would reduce the power and generalizability. Secondly, some of the therapy materials are culturally specific, e.g., photos of celebrities/places, stimuli used in word games, etc. The generalization of the findings to other cultures should be made with caution. Finally, other limitations, including MCI participants not being blinded to therapy conditions and the lack of long-term follow-up (6-month or longer), also exist. Nonetheless, the initial findings will inform us about the feasibility of IVR-CST, and the efficacy will help us decide if more sources should be devoted to scaling up the study.

### Dissemination plan

It is planned that in four to six months, upon completion of data collection and preliminary data analyses, the initial results will be disseminated in local and overseas conferences on topics such as dementia intervention and management, ageing, and the use of VR or other advanced technologies in rehabilitation. The first author, Wong, who is also the principal investigator of the study, will lead the co-authors in preparing detailed reports, which will be submitted to international peer-reviewed journals six months after completion of data analysis. Last but not least, workshops introducing IVR-CST in terms of its technical requirements, set-up, and clinical efficacy will be held face-to-face/online for healthcare professionals and caregivers supporting dementia care and rehabilitation in Hong Kong and neighboring cities. The anonymized dataset will be accessible via an open-access data repository.

## Supporting information

S1 Table SPIRIT checklist. Details of the current study protocol in SPIRIT checklist.
(DOCX)

## References

[1] Cooper C, Li R, Lyketsos C, Livingston G. Treatment for mild cognitive impairment: systematic review. Br J Psychiatry. 2013;203(3):255–64. doi: 10.1192/bjp.bp.113.127811 24085737 PMC3943830

[2] Ward A, Arrighi HM, Michels S, Cedarbaum JM. Mild cognitive impairment: disparity of incidence and prevalence estimates. Alzheimers Dement. 2012;8(1):14–21. doi: 10.1016/j.jalz.2011.01.002 22265588

[3] Xu Z, Zhang D, Sit RWS, Wong C, Tiu JYS, Chan DCC, et al. Incidence of and Risk factors for Mild Cognitive Impairment in Chinese Older Adults with Multimorbidity in Hong Kong. Sci Rep. 2020;10(1):4137. doi: 10.1038/s41598-020-60901-x 32139719 PMC7057945

[4] Tschanz JT, Welsh-Bohmer KA, Lyketsos CG, Corcoran C, Green RC, Hayden K, et al. Conversion to dementia from mild cognitive disorder: the Cache County Study. Neurology. 2006;67(2):229–34. doi: 10.1212/01.wnl.0000224748.48011.84 16864813

[5] Fitzpatrick-Lewis D, Warren R, Ali MU, Sherifali D, Raina P. Treatment for mild cognitive impairment: a systematic review and meta-analysis. CMAJ Open. 2015;3(4):E419-27. doi: 10.9778/cmajo.20150057 26770964 PMC4701654

[6] Gómez-Soria I, Marin-Puyalto J, Peralta-Marrupe P, Latorre E, Calatayud E. Effects of multi-component non-pharmacological interventions on cognition in participants with mild cognitive impairment: A systematic review and meta-analysis. Arch Gerontol Geriatr. 2022;103:104751. doi: 10.1016/j.archger.2022.104751 35839574

[7] Chu C-S, Li C-T, Brunoni AR, Yang F-C, Tseng P-T, Tu Y-K, et al. Cognitive effects and acceptability of non-invasive brain stimulation on Alzheimer’s disease and mild cognitive impairment: a component network meta-analysis. J Neurol Neurosurg Psychiatry. 2021;92(2):195–203. doi: 10.1136/jnnp-2020-323870 33115936 PMC7841477

[8] Clare L, Woods RT. Cognitive training and cognitive rehabilitation for people with early-stage Alzheimer’s disease: a review. Neuropsychol Rehabil. 2004;14:385–401.

[9] Woods B, Rai HK, Elliott E, Aguirre E, Orrell M, Spector A. Cognitive stimulation to improve cognitive functioning in people with dementia. Cochrane Database Syst Rev. 2023;1(1):CD005562. doi: 10.1002/14651858.CD005562.pub3 39804128 PMC9891430

[10] NICE National Institute of Health and Care Excellence. Dementia: Assessment, Management and Support for People Living with Dementia and Their Carers. London: National Institute of Health and Care Excellence. 2018. https://www.nice.org.uk/guidance/ng97/evidence 30011160

[11] Spector A, Thorgrimsen L, Woods B, Royan L, Davies S, Butterworth M, et al. Efficacy of an evidence-based cognitive stimulation therapy programme for people with dementia: randomised controlled trial. Br J Psychiatry. 2003;183:248–54. doi: 10.1192/bjp.183.3.248 12948999

[12] Wong GHY. Making a difference: an evidence-based group program to offer cognitive stimulation therapy to people with dementia; the manual for group leaders. Hong Kong: Hong Kong University Press. 2017.

[13] Wong GHY, Yek OPL, Zhang AY, Lum TYS, Spector A. Cultural adaptation of cognitive stimulation therapy (CST) for Chinese people with dementia: multicentre pilot study. Int J Geriatr Psychiatry. 2018;33(6):841–8. doi: 10.1002/gps.4663 29717527

[14] Hui EK, Wong GHY, Tischler V, Yuan SNV, Leung WG, Saunders R, et al. Virtual individual cognitive stimulation therapy in Hong Kong: A mixed methods feasibility study. Geriatr Nurs. 2022;47:125–34. doi: 10.1016/j.gerinurse.2022.07.010 35908368

[15] Haroutunian V, Hoffman LB, Beeri MS. Is there a neuropathology difference between mild cognitive impairment and dementia?. Dialogues Clin Neurosci. 2009;11(2):171–9. doi: 10.31887/DCNS.2009.11.2/vharoutunian 19585952 PMC3073531

[16] Gomez-Soria I, Peralta-Marrupe P, Plo F. Cognitive stimulation program in mild cognitive impairment A randomized controlled trial. Dement Neuropsychol. 2020;14(2):110–7. doi: 10.1590/1980-57642020dn14-020003 32595879 PMC7304274

[17] Gómez-Cáceres B, Cano-López I, Aliño M, Puig-Perez S. Effectiveness of virtual reality-based neuropsychological interventions in improving cognitive functioning in patients with mild cognitive impairment: A systematic review and meta-analysis. Clin Neuropsychol. 2023;37(7):1337–70. doi: 10.1080/13854046.2022.2148283 36416175

[18] Tortora C, Di Crosta A, La Malva P, Prete G, Ceccato I, Mammarella N, et al. Virtual reality and cognitive rehabilitation for older adults with mild cognitive impairment: A systematic review. Ageing Res Rev. 2024;93:102146. doi: 10.1016/j.arr.2023.102146 38036103

[19] Liao Y-Y, Tseng H-Y, Lin Y-J, Wang C-J, Hsu W-C, Liao YY, et al. Using virtual reality-based training to improve cognitive function, instrumental activities of daily living and neural efficiency in older adults with mild cognitive impairment. Eur J Phys Rehabil Med. 2020;56(1):47–57. doi: 10.23736/S1973-9087.19.05899-4 31615196

[20] Sayma M, Tuijt R, Cooper C, Walters K. Are We There Yet? Immersive Virtual Reality to Improve Cognitive Function in Dementia and Mild Cognitive Impairment. Gerontologist. 2020;60(7):e502–12. doi: 10.1093/geront/gnz132 31600389

[21] Oliveira J, Gamito P, Souto T, Conde R, Ferreira M, Corotnean T, et al. Virtual Reality-Based Cognitive Stimulation on People with Mild to Moderate Dementia due to Alzheimer’s Disease: A Pilot Randomized Controlled Trial. Int J Environ Res Public Health. 2021;18(10):5290. doi: 10.3390/ijerph18105290 34065698 PMC8156930

[22] Wong WWS, Tsang HT, Luk CL, Chiu ATS. Effects of applying Virtual Reality in Cognitive Stimulation Therapy on cognition and functional communication of people with concurrent dementia and visual impairment: An initial Study [Poster presentation]. The Alzheimer’s Association International Conference 2023, Amsterdam.

[23] Adhanom IB, MacNeilage P, Folmer E. Eye tracking in virtual reality: a broad review of. 2023.Adhanom IB, MacNeilage P, Folmer E. Eye Tracking in Virtual Reality: a Broad Review of Applications and Challenges. Virtual Real. 2023;27(2):1481–505. doi: 10.1007/s10055-022-00738-z 37621305 PMC10449001

[24] Liu Z, Yang Z, Gu Y, Liu H, Wang P. The effectiveness of eye tracking in the diagnosis of cognitive disorders: A systematic review and meta-analysis. PLoS One. 2021;16(7):e0254059. doi: 10.1371/journal.pone.0254059 34252113 PMC8274929

[25] Chiu HFK, Zhong B-L, Leung T, Li SW, Chow P, Tsoh J, et al. Development and validation of a new cognitive screening test: The Hong Kong Brief Cognitive Test (HKBC). Int J Geriatr Psychiatry. 2018;33(7):994–9. doi: 10.1002/gps.4883 29642275

[26] Kennedy RS, Lane NE, Berbaum KS, Lilienthal MG. Simulator Sickness Questionnaire: An Enhanced Method for Quantifying Simulator Sickness. The International Journal of Aviation Psychology. 1993;3(3):203–20. doi: 10.1207/s15327108ijap0303_3

[27] Yeung PY, Wong LLL, Chan CC, Yung CY, Leung LMJ, Tam YY, et al. Montreal Cognitive Assessment - Single Cutoff Achieves Screening Purpose. Neuropsychiatr Dis Treat. 2020;16:2681–7. doi: 10.2147/NDT.S269243 33192067 PMC7656779

[28] Kirova A-M, Bays RB, Lagalwar S. Working memory and executive function decline across normal aging, mild cognitive impairment, and Alzheimer’s disease. Biomed Res Int. 2015;2015:748212. doi: 10.1155/2015/748212 26550575 PMC4624908

[29] McDonnell M, Dill L, Panos S, Amano S, Brown W, Giurgius S, et al. Verbal fluency as a screening tool for mild cognitive impairment. Int Psychogeriatr. 2020;32(9):1055–62. doi: 10.1017/S1041610219000644 31258101 PMC9153280

[30] Junquera A, García-Zamora E, Olazarán J, Parra MA, Fernández-Guinea S. Role of Executive Functions in the Conversion from Mild Cognitive Impairment to Dementia. J Alzheimers Dis. 2020;77(2):641–53. doi: 10.3233/JAD-200586 32741835

[31] Leung SOC, Chan CCH, Shah S. Development of a Chinese version of the Modified Barthel Index-- validity and reliability. Clin Rehabil. 2007;21(10):912–22. doi: 10.1177/0269215507077286 17981850

[32] Tong AYC, Man DWK. The Validation of the Hong Kong Chinese Version of the Lawton Instrumental Activities of Daily Living Scale for Institutionalized Elderly Persons. OTJR: Occupational Therapy Journal of Research. 2002;22(4):132–42. doi: 10.1177/153944920202200402

[33] Krueger C, Tian L. A comparison of the general linear mixed model and repeated measures ANOVA using a dataset with multiple missing data points. Biol Res Nurs. 2004;6(2):151–7. doi: 10.1177/1099800404267682 15388912

[34] Bellg AJ, Borrelli B, Resnick B, Hecht J, Minicucci DS, Ory M, et al. Enhancing treatment fidelity in health behavior change studies: best practices and recommendations from the NIH Behavior Change Consortium. Health Psychol. 2004;23(5):443–51. doi: 10.1037/0278-6133.23.5.443 15367063

